# Prediction of Rowing Ergometer Performance from Functional Anaerobic Power, Strength and Anthropometric Components

**DOI:** 10.2478/hukin-2014-0041

**Published:** 2014-07-08

**Authors:** Fırat Akça

**Affiliations:** 1 Ankara University, Faculty of Sport Sciences, Ankara/TURKIYE.

**Keywords:** performance prediction model, simulated rowing, talent identification

## Abstract

The aim of this research was to develop different regression models to predict 2000 m rowing ergometer performance with the use of anthropometric, anaerobic and strength variables and to determine how precisely the prediction models constituted by different variables predict performance, when conducted together in the same equation or individually. 38 male collegiate rowers (20.17 ± 1.22 years) participated in this study. Anthropometric, strength, 2000 m maximal rowing ergometer and rowing anaerobic power tests were applied. Multiple linear regression procedures were employed in SPSS 16 to constitute five different regression formulas using a different group of variables. The reliability of the regression models was expressed by R2 and the standard error of estimate (SEE). Relationships of all parameters with performance were investigated through Pearson correlation coefficients. The prediction model using a combination of anaerobic, strength and anthropometric variables was found to be the most reliable equation to predict 2000 m rowing ergometer performance (R2 = 0.92, SEE= 3.11 s). Besides, the equation that used rowing anaerobic and strength test results also provided a reliable prediction (R2 = 0.85, SEE= 4.27 s). As a conclusion, it seems clear that physiological determinants which are affected by anaerobic energy pathways should also get involved in the processes and models used for performance prediction and talent identification in rowing.

## Introduction

Competitive rowing is a sport discipline that requires highly developed aerobic and anaerobic capacity. The energy needed for a 2000 m ergometer rowing was estimated to be 65–75% aerobic and 25–35% anaerobic (Droghetti et al., 1991). Anaerobic power is specifically important during the initial spurt and final dash and characteristically ensures 20–30% of the energy requirement of a 2000 m race (Secher, 1993). Due to a large contribution of anaerobic metabolic processes, efficiency of anaerobic energy pathways may also be a significant predictor of rowing performance.

Although researchers and coaches have a chance to measure each rower’s power output independently on the water with the help of recently developed devices which calculate power output from kinematic data measured by sensors in rowlock and/or oar(s), these devices are expensive, time-consuming to install and calibrate, and often fragile. Standardisation problems, caused by environmental conditions mostly during on-water testing, led researchers to the widespread use of rowing ergometers that simulate the action of on-water rowing. Some differences have been found in particular rowing ergometry studies in arm motion, handle force and acceleration profiles and consistency in stroke timing between off-water and on-water rowing performance. Despite these differences, the 2000 m rowing ergometer time trial is the most common measure of rowing performance. Measures of performance derived from rowing ergometer tests do have standard error of measurement less than 1% in reliability studies. The 2000 m time trial on the rowing ergometer has become an important selection tool for national rowing organizations (Smith and Hopkins, 2012). By testing rowers individually through competition-length time trials on ergometers, coaches are able to evaluate each athlete’s ranking on the team in a controlled environment. When comparing on-water to simulated rowing methodologies only slight discrepancies in physiological responses exist (Izquierdo-Gabarren et al., 2010; Vogler et al., 2010). Nevill et al. (2010) determined that dividing the rowing ergometer speed by body mass would greatly improve the ability of ergometer performance to accurately reflect rowing performance on water.

A favourable anthropometric profile may be considered as an important factor to athlete’s performance in addition to the technique of movement and experience. Once identified, these attributes may be used for talent identification and to develop a more specific assessment. Furthermore, it may assist the coach or sport scientists in constructing a training program that enhances all of the essential attributes to the levels required for success. Several papers show that anthropometric characteristics may have some effect on rowing performance. Rowers with better results are likely to be taller (Hahn, 1990) and heavier (Secher et al., 1983), and further they have lower skinfold values than less successful competitors. Many have long extremities, not only in absolute terms but also in proportion to their standing height (Hahn, 1990).

Performance prediction regression analyses models have already been properly established for road cycling (Coyle et al., 1988), track cycling (Craig et al., 1993), and distance running (Morgan et al., 1989) employing both anthropometric and metabolic parameters, such as body mass, sum of skinfolds, oxygen uptake (VO_2max_), anaerobic capacity, blood lactate thresholds and exercise economy. Russell et al. (1998) came to the conclusion that prediction models formulated from both anthropometric factors or a combination of somatic and physiological variables are more effective predictors of 2000 m performance than any single variable. There are few anaerobic tests especially developed to evaluate power of rowers (Mandic et al., 2004; Riechman et al., 2002). The Wingate sprint test using a rowing ergometer reported high trial-to-trial accuracy for average and maximum power (Riechman et al., 2002). In the same study, they determined that rowing ergometer performance of lightweight and heavyweight female rowers could be predicted reliably using the Wingate rowing test mean power, fatigue index and VO_2max_.

Previous research projects were conducted on rowers with different abilities, yet, there is no prediction study in the literature which concerns collegiate level rowers. The diversity of variables employed in the prediction models developed earlier is limited. In this study more detailed analyses were accomplished and more variables were employed to shape performance prediction models compared to other studies which related to rowing performance prediction equations.

The aim of the present study was to develop different regression models to predict 2000 m rowing ergometer performance. Various anthropometric, anaerobic and strength variables were employed to shape the prediction formulas. The best possible combination of employed variables which gave the best prediction result was demonstrated with statistical details.

## Material and Methods

### Participants

Thirty-eight male collegiate rowers (age= 20.17±1.22 years) voluntarily participated in this study. After securing institutional ethical approval, participants were provided with a sheet which contained information with regard to the study design and any possible risks, to ensure that they were familiar with the procedures and that they could withdraw from the study at any time. After providing their written informed consent, a health screening (PAR-Q assessment) was completed. Participants were asked to refrain from any food intake for three hours before the measurements and to avoid caffeine, alcohol and strenuous exercise for 48 hours before tests.

### Procedures

Variables, which showed a significant correlation with the rowing performance, were employed to constitute regression models. Five different regression models were constituted and predictive ability of each model was compared in order to identify the most precise model. To test the hypothesis developed, participants visited the laboratory on four different occasions. The interval between each testing day was at least three days. Within two weeks, participants completed anthropometric measures at visit 1, strength measurements at visit 2, anaerobic power measures at visit 3 and an all-out 2000m rowing ergometer test at visit 4.

### Anthropometry

Measurements were carried out in accordance with standard anthropometric techniques recommended by Norton and Olds (2004). Skinfold measurements were taken with a Holtain (UK) caliper at the following sites: biceps, triceps, subscapular, suprailiac, calf, abdomen, thigh and chest. In addition, humerus biepicondylar, femur biepicondylar and biacromial widths were measured as well as the girths of the flexed and tensed biceps, calf, thigh, forearm and upper arm. Arm span, leg and arm lengths were also measured.

The median was used in statistical analysis if the measurements had to be taken three times, while the mean was utilized if the first two measurements were within the acceptable range (5%) (Norton and Olds, 2004). The Heath-Carter method was used to estimate somatotype (Carter and Heath, 1990). The body fat percentages of athletes were estimated according to the method of Yuhasz (1990). Lean body mass of the participants was measured using a Tanita TBF 410 MA (Tokyo, Japan) body composition analyser (Boneva-Asiova and Boyanov, 2008).

### Arm Strength

Arm strength of participants was measured by the biceps strength test (Polar Trifit 700 fitness assessment system, Polar Electro, NY, USA). During the test, participants stood with their backs straight and legs tight, and held the dynamometer with their palms facing upward and the angle of elbow at 90 degrees, they applied maximum strength for five seconds. Two more trials were performed with three-minute rest intervals after each trial and the best result was recorded.

### Leg Strength

Leg strength of the participants was measured through a 1 repetition maximum leg press (1 RM) test. During the test, participants grasped the handle of the seat while maintaining a straight back. Also participants placed their feet on the machine footings and they were required to flex their knees to 90 degrees. Individuals were instructed to warm-up with a light weight for 5 repetitions. Following a three-minute rest period, a weight was estimated to allow 3 repetitions. Weights were increased as necessary until a 1 RM was determined. Three- minute rest periods followed each set. If the participant failed, the load was decreased by 5–10 kg for the next trial. By increasing or decreasing the load, the participants were able to complete a 1 RM within five sets. The maximum load with which a repetition was performed successfully was used for data analysis.

### Back Strength

Back strength of the participants was measured through a 1 RM test bench pull. Bench-pull (elbow and shoulder flexion) was selected due to the fact that it seems to be the most specific exercise to the rowing technique (McNeely et al., 2005). Bilateral bench pull tests were accomplished by using standard bench pull equipment with the participants adopting a position (lying face down on the bench with their arms completely stretched out and hands holding on to the bar), and with their weight suspended perpendicularly at 90°. A manual goniometer (Moeltgen, Hillside Supplies Ltd., Notts, UK) was employed to the elbow to standardize the range of motion. On command, the participants performed a concentric arm flexion beginning from the extended position to reach full flexion (touching the bench) against the resistance determined by the weight. The warm-up was comprised of a set of 10 repetitions with loads of 40–60% of the perceived maximum. Thereafter, five to six separate single attempts were made until the subject was unable to bend the arms into the required position. The last acceptable flexion with the highest possible load was determined as 1RM. Three minute rest periods were given between trials.

### Anaerobic Power Test

A modified Wingate test, using a Concept II model C rowing ergometer (Concept II, Morrisville, VT, USA) was used to measure functional anaerobic power (Riechman et al., 2002).

Participants were instructed to stretch and warm up on the ergometer for 3 minutes at moderate intensity (approximately 60%). After the warm up, the ergometer was programmed for a 30 s trial at the maximum damper setting (10 on the resistance control dial). The participants then completed an “all-out” 30 s effort with verbal encouragement. Power was calculated and displayed on an ergometer screen for every stroke and recorded for each stroke.

Mean power was described as the average of individual stroke power over the 30 s trial. Maximal power was described as the mean of the five highest consecutive strokes. In addition, minimal power was described as the mean of the five lowest consecutive strokes after attainment of maximal power. The fatigue index was calculated upon the percent difference in maximal and minimal power.

### 2000 m. Maximal Rowing Ergometer Test

The participants were asked to perform an allout 2000 m test on a rowing ergometer. The screen of an ergometer was set to display remaining metres, average 500 m time and accumulated time. Verbal encouragement was given during the last 250 meters of the test. Heart rate (HR) was recorded with a telemetric HR monitor throughout the test (Polar RS 400, Polar Electro, Kempele, Finland). The performance feedback viewed by participants from the ergometer’ screen was 500 m average time, total time and the distance remaining. Completion time, stroke rate, HR, average power outputs were recorded immediately after the test for the whole test and each 500 m splits separately.

### Statistical Analysis

Enter type regression analyses were employed to constitute five different prediction models which use anthropometric measurement results, strength test results, anaerobic power test results, combination of the strength and anaerobic power and combination of all measurement categories. For all equations, the variables which resulted in a lowest possible standard error of estimate (SEE) were used. Reliability of the regression models was expressed by R^2^ and the SEE. The adjusted R^2^, as opposed to the sample R^2^, was used to assess the proportion of variance that could be explained by the independent variables. According to Russell et al. (1998), the adjusted R^2^ considers both the number of predictor variables and the sample size.

This results in the R^2^ being approximately corrected for the upward bias of the sample R^2^, which subsequently provides a more accurate estimate of goodness of fit into the prediction model. The statistical power of the applied analysis ranged from 0.90 to 0.99 in all cases. To determine the relationships with performance of all measured parameters the Pearson product correlation coefficient was used. The level of significance was set at p < 0.05. All data was analysed using the SPSS 16 statistics software (Chicago, IL, USA).

## Results

General physical characteristics of the participants and their correlation with 2000 m rowing performance are presented in Table 1. Performance was significantly correlated with body height (r = −0.801), body mass (r = −0.812), lean body mass (r = −0.822) and sitting height (r = −0.687).

Girth, width and length values of participants are presented in Table 2. Performance was significantly correlated with forearm girth (r = −0.615), upper arm girth (r = −0.629), flexed biceps girth (r = −0.655), calf girth (r = −0.5550), thigh girth (r = −0.694), biacromial width (r = −0.631), femur width (r = −0.678), humerus width (r = −0.665), arm span (r = −0.715), arm length (r = −0.701) and leg length (r = −0.703).

Anaerobic power and strength characteristics of the participants and their correlation with 2000 m rowing performance are presented in Table 2. Performance was significantly correlated with Wingate test mean power (r = −0.796), maximum power (r = −0.756), minimum power (r = −0.778), leg press 1 RM (r = −0.755), bench pull 1 RM (r = −0.749) and biceps strength (r = −0.728).

The rowing ergometer performance prediction models using different categories of variables are presented in Table 3.

## Discussion

According to the results of the present study body height and body mass were significantly correlated with rowing ergometer performance. These findings were consistent with previous studies by Kramer et al. (1994) and Cosgrove et al. (1999). Malina (1994) observed that promising rowers were already taller compared to the general population within childhood, and they retained their relative advantage during adolescence. Shephard (1998) noted that gold medallists were consistently taller and heavier than the other competitors; in the event of the single sculls, the particular variances were significant 0.12 m and 9.6 kg, respectively.

When Wingate anaerobic test and strength test results were taken into account, important relationships with the performance were noted. They were in line with the studies of Russell et al. (1998) and Riechman et al. (2002) who reported statistically significant correlations in the range of r = 0.84 to r = 0.89 between the Wingate rowing anaerobic test results and 2000 m rowing ergometer performance. The significant relationship between the Wingate test results and the 2000 m rowing ergometer performance is probably explained, in part by a substantial aerobic contribution to a 30 s all-out test. Additionally, Secher (1993) indicated that the `initial spurt’ accomplished at the beginning of rowing events may be crucial for optimal performance and may be dependent on the maximal anaerobic capacity of the oarsman. Riechman et al. (2002) employed multiple regression procedures to predict 2000 metre performance in rowers. The Equation has the prediction power of R^2^ = 0.757 and SEE = 6.37 and the one and only variable that was used in the equation was the Wingate test average power. These results are very similar to the reliability of the prediction equation that was constituted byusing Wingate rowing anaerobic power test variables in this study.

Chun Jung et al. (2007) reported significant relationships between the leg press and inverted row values and 2000 m rowing ergometer performance. The results of the present study also showed significant relationships between strength values and performance. The contradictory reports of comparatively high strength in rowers and the deficiency of predictive value of these variables (Secher, 1975) are likely to be the consequence of evaluations which are not mode specific (such as Wingate arm-crank, Wingate cycle test, isokinetic strength) or test procedures that rely on aerobic metabolism; such as accumulated oxygen deficit (Russell et al., 1998). Precise performance prediction model has been developed using strength variables in the present study, thus effects of strength on rowing performance should be of greater focus in future studies.

Using Wingate anaerobic test results and 1 RM strength test results seem logical to predict 2000 m rowing ergometer performance according to results of the present study. These test protocols are easy to apply for coaches and athletes and not time consuming. Employing simpler equations instead of the overall, combined equation would be a good alternative as they do not require expensive laboratory equipment, trained staff, and can be completed fairly quickly.

The correlation between performance time and anthropometric equation was high in the present study. Russell et al. (1998) indicated that a prediction model, developed from anthropometric variables, was a good predictor of 2000 m ergometer performance. They found adjusted R value as r = 0.78 in their anthropometric equation similar to the one in the present study. Jurimae et al. (2000) constructed different prediction models to predict 2500 m rowing ergometer performance, they indicated that the prediction model which used the combination of physiological measures predicted rowing performance best (R = 0.99), followed by metabolic (R = 0.99) and anthropometric (R = 0.76) variables in lightweight rowers. In open class rowers, the best prediction model was found to be a combination of physiological measures (R = 0.82), followed by anthropometric (R = 0.76) and metabolic (R = 0.70) variables.

The high correlation between the anthropometric equation and 2000 m performance time in the present study may be explained by the relationship between morphology and human performance. Carter and Heath (1990) revealed that the morphology was associated with both the physiology and biomechanics of humans in motion. As determined in numerous studies, a typical heavyweight rower’s morphological phenotype represents a tall, heavy and lean athlete with a high percentage of slow-twitch muscle fibres. These morphological characteristics occur directly from the specific rowing training and genetic inheritance. The large volume of aerobic training undertaken, together with weight training provides a rower with a high aerobic power, enhanced skill and metabolic efficiency, low skinfolds and a greater muscle mass. In support of this finding, Hahn (1990) indicated that more successful rowers are tall, heavy and possess a low skinfold reading. Bourgois et al. (2000) reported that rowers who competed in the final of world junior rowing championships had significantly higher values for length, width and girth dimensions than non-finalists. The situation is similar in lightweight rowers so Rodriguez (1986) demonstrated that the medallists in lightweight rowing had significantly higher length, girth and width values than nonmedallists. It appears obvious that the evaluation of rowers should contain several anthropometric attributes.

The main objective of producing rowing performance prediction models is to use statistical methods to specify essential performance limiting physiological elements that can be particularly trained to maximize performance. The use of laboratory-based prediction models help coaches to predict on-water rowing performance and to determine potentially talented rowers. Rodriguez et al. (1990) revealed that rowing ergometry duplicated the kinematic movement structures of, and yields identical VO_2max_ values to, on-water rowing. Nevertheless, personal rowing ergometer performance times do not account for the differences in skill and efficiency when rowing as a team on the water; therefore, caution is suggested when using these performance models to predict on-water rowing performance. Sparrow and Newell (1994) stated that skilled performance in repeating gross motor exercises, like rowing, is related to the economy of metabolic energy expenditure.

These results (prediction equations) must be viewed with caution, as the prediction equations were developed particularly for male collegiate rowers. The prediction equations established are only as accurate as the tests used to measure the anthropometric, anaerobic and strength variables. The prediction variables identified in this study may be specific to the sample of collegiate rowers. The use of these predictors in a wide range of rowers, together with cross-validation in an independent sample of rowers is necessary.

The present results are in line with other studies, suggesting that strength, anaerobic power and anthropometry are important training objectives to optimize 2000 m rowing performance. In addition, the methods used in the current research produced statistical models using anthropometric, anaerobic, strength, anaerobic-strength together and the combination of all variables. The created equations were capable of precise predicting 2000 m rowing ergometer performance in a group of competitive male collegiate rowers. It may be possible to develop similar statistical models for other competitive sports using various sport-specific testing variables.

The predictive values of the variables presented in this study can be seen as an important addition to the talent identification purposes of rowing. When relationships determined between height, body mass, lean mass and performance in the present study are taken into account, using the variables discussed above in the initial stages of the talent identification process could be logical. Rowing Wingate and strength test results can be used in latter stages of the talent identification process since performance in these tests can be effected by movement technique and athletic experience.

Strong relationships between certain anthropometric, anaerobic and strength measures and performance were observed. Therefore, the effects of physical structure, anaerobic capacity and strength during the rowing performance should not be underestimated. Anaerobic, anthropometric and strength components must be included in the rowing performance prediction formulas.

## Figures and Tables

**Table 1:** General physical characteristics and 2000 m performance of the participants

**Variables**	**Mean**	**S.D**	**Min.**	**Max.**	**r (2000 m)**
Body Height (cm)	185.77	9.14	173.8	194.6	−.801^**^
Body Mass (kg)	80.23	9.22	69,7	90.8	−.812^**^
Body Mass Index	22.42	0.84	20	24.85	−.296
2000 meters time (s)	398.50	20.11	371.2	422.8	1
Lean Mass (kg)	62.21	5.55	52.35	73.81	−.822^**^
Body Fat Percentage (%)	10.02	0.92	6.75	13.11	−.185
Sitting Height (cm)	96.99	1.75	92.53	98.44	−.687^**^
Endomorphy	2.89	0.55	1.78	3.82	−.191
Mesomorphy	4.72	0.88	3.25	6.11	−.503
Ectomorphy	3.01	0.49	2.22	4.66	−.197

* p < 0.05;

** p < 0.01

**Table 2:** Anthropometric, anaerobic and strength characteristics of the participants and correlations with 2000 m rowing performance

**Variables**	**Mean**	**S.D**	**Min.**	**Max.**	**r (2000 m.)**
Forearm girth (cm)	30.01	2.51	26.1	32.12	−.615^*^
Upper arm girth (cm)	29.91	2.59	28.0	33.00	−.629^*^
Flexed Biceps girth (cm)	33.13	2.77	30.7	36.0	−.655^**^
Calf girth (cm)	38.08	3.02	34.7	41.3	−.550^*^
Thigh girth (cm)	58.35	4.25	55.1	63.2	−.694^**^
Biacromial width (cm)	40.98	1.77	38.9	45.2	−.631^*^
Femur width (cm)	9.87	0.66	8.7	10.9	−.678^**^
Humerus width (cm)	7.39	0.81	6.0	8.6	−.665^**^
Arm span (cm)	188.44	8.85	175.1	201.4	−.715^**^
Arm length (cm)	83.86	3.49	75.1	87.8	−.701^**^
Leg Length (cm)	90.67	5.88	85.1	95.8	−.703^**^
Wingate test mean power (W)	638	41.80	539	702	−.796^**^
Wingate test maximum power (W)	659	58.11	544	720	−.756^**^
Wingate test minimum power (W)	563	57.16	493	656	−.778^**^
Fatigue index (%)	15.8	4.9	6.62	21.95	.283
Leg press 1 RM (kg)	181.85	25.55	140	230	−.755^**^
Bench pull 1 RM (kg)	95.90	12.20	70	130	−.749^**^
Biceps strength (kg)	65.66	6.11	60	85	−.728^**^

* p < 0.05;

** p < 0.01

**Table 3:** Multiple regression equations, R^2^, variance and standard error of estimate (SEE) for different categories

**Category**	**Multiple regression equation**	**R^2^ Variance SEE**
**Anthropometric**	2000 m time (s) = 864.127+(−3.585 × sitting height)+(−1.005 × lean body mass)+(0.566 × arm span)+(−0.885 × body height)+(−0.365 × thigh girth)+(−0.212 × body mass)+(0.235 × leg length)+(0.244 × arm length)+4.71	0.83 77% 4.71
**Strength**	2000 m time (s) = 654.314 + (−0.864 × body height) + (−0.238 × bench pull 1 RM) + (−0.676 × biceps 1 RM) + (−0.052 × leg press 1 RM) + (−0.141 × weight) + 5.29	0.80 74% 5.29
**Anaerobic Power**	2000 m time (s) = 611.317+(−0.511 × body height)+(−0.568 × body mass)+ (−0.059 × wingate test average power)+(−0.011 × Wingate test max. power)+(−0.010 × wingate test min. power) + 6.27	0.76 70% 6.27
**Anaerobic-Strength**	2000 m time (s) = 638.185 + (−0.866 × body height) + (−0.319 × bench pull 1 RM) + (−0.114 × leg press 1 RM) + (−0.146 × body mass) + (−0.017 × Wingate test average power) + (−0.002 × Wingate test min. power) + 4.27	0.85 80% 4.27
**Combination of all**	2000 m time (s) = 611.059 + (−1.046 × lean body mass) + (−1.762 × body height) + (−0.521 × bench pull 1 RM) + (0.313 × arm length) + (0.674 × leg length) + (0.461 × weight) + (0.309 × armspan) + (−0.411 × Wingate test max. power) + (−0.57 × Wingate test min. Power) + (0.044 × Wingate test average power) + (−0.011 × leg press 1 RM) + 3.11	0.92 87% 3.11
